# Salvaging Percutaneously a Suboptimal TAVR Deployment with Repositioning In the Thoracic Aorta: A Novel Snare-Mediated Approach to Prosthesis Malposition

**DOI:** 10.1016/j.jaccas.2025.104553

**Published:** 2025-07-18

**Authors:** Sandeep M. Patel, Hafez Golzarian, Anna C. Kleman, Mallory Knous, Sarah L. Kallay, Sarah E. Malloy, Maile A. Miller, Emilie Scarbrough, Gerri Hempfling, John Sirak

**Affiliations:** a Structural Heart & Intervention Center, Mercy Health—St. Rita's Medical Center, Lima, Ohio, USA; b Internal Medicine Residency Program, Mercy Health—St. Rita's Medical Center, Lima, Ohio, USA; c Cardiovascular Disease Fellowship Program, HCA Houston Healthcare – Kingwood/University of Houston College of Medicine, Houston, Texas, USA; d Department of Cardiothoracic Surgery, Mercy Health—St. Rita's Medical Center, Lima, Ohio, USA

**Keywords:** aortic valve replacement, bioprosthesis, heart failure, paravalvular leak, snare, valve-in-valve

## Abstract

**Objective:**

To highlight a novel and strategic approach to managing a malpositioned transcatheter aortic valve replacement valve using a double-snare–assisted reimplantation strategy.

**Key Procedural Steps:**

Key procedural steps include maintaining guidewire access through the valve, using a True Flow balloon to enhance control over valve maneuverability and repositioning, applying a crisscross double-snare technique allowing the valve to fold inward and minimize aortic trauma, carefully repositioning the valve to a desirable location in the aorta, maintaining control of the mobilized valve with a snare, and finally, properly implanting a new valve.

**Potential Pitfalls:**

Potential risks and complications of the procedure include aortic trauma, stroke, valve entanglement, and undesirable valve embolization as well as the possible need for conversion to open heart surgery.

**Take-Home Messages:**

The double-snare–assisted repositioning technique may offer a feasible percutaneous approach for managing malpositioned transcatheter aortic valve replacement valves. Given the increasing frequency of high-risk valve-in-valve procedures, clinicians should aim to enhance their expertise in managing these challenging complications.

## Graphical Abstract

**Figure:**
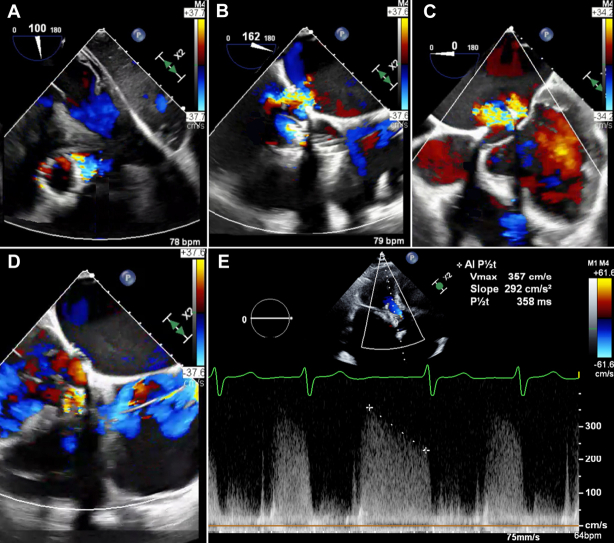

The procedural efficacy of transcatheter aortic valve replacement (TAVR) has advanced significantly in the past decade; however, valve malposition persists as a prevalent complication, affecting approximately 3% of cases.^1^ In the event of TAVR malpositioning, the standard approach to management is urgent implantation of another valve during or shortly after the procedure. Nonetheless, in a small number of instances, the sole deployment of an additional TAVR valve may prove insufficient. We present a case of an elderly patient with multiple comorbidities and prohibitive surgical risk who presented 3 weeks after undergoing a valve-in-valve TAVR. The valve positioning was suboptimal, resulting in symptomatic severe paravalvular leak and interference with the mitral valve. We were able treat the patient by meticulously snaring the valve and intentionally repositioning it proximally in the ascending aorta distal to the innominate artery, which then enabled us to properly reimplant a new valve. We discuss the snare technique used to address this deep-rooted challenge as well as its clinical implications.

**Take-Home Messages**

• The double-snare–assisted reimplantation strategy may be a viable percutaneous salvage strategy for patients with malpositioned TAVR valves. With the growing prevalence of high-risk valve-in-valve procedures, proceduralists should strive to refine their skillset to effectively address these complications.
• Key technical aspects to ensure controlled valve extraction and repositioning include maintaining guidewire access through the valve, using a double-snare technique for stability, using a True Flow balloon for maneuverability and to minimize aortic trauma, and proper patient selection.

## Case Summary

**Figure 1:**
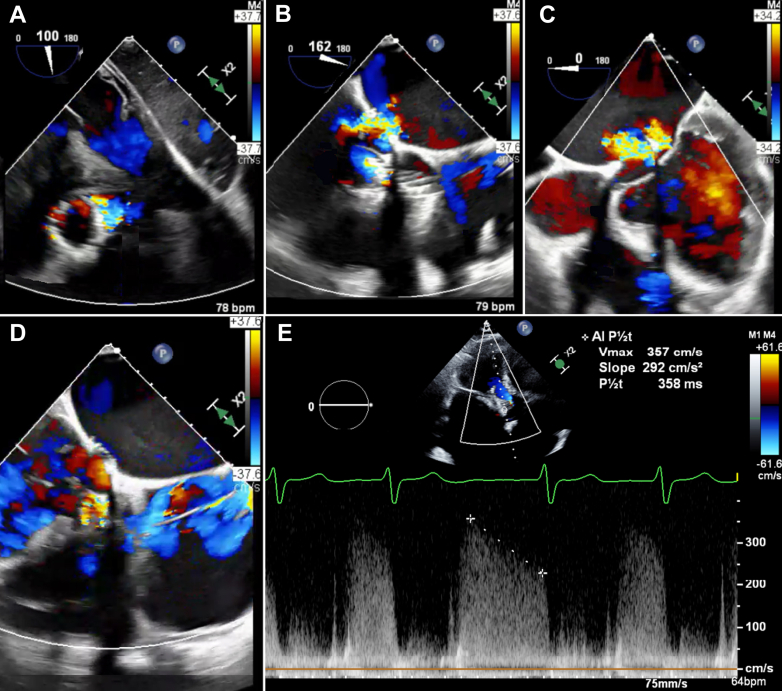
Transesophageal Echocardiogram Findings (A) Paravalvular leak (short axis). (B) Paravalvular leak (long axis). (C) Mitral regurgitation (midesophageal 4-chamber). (D) Mitral regurgitation and restriction of the anterior mitral leaflet (midesophageal reverse 4-chamber). (E) Regurgitant flow waveform (color Doppler).

An 85-year-old man with history of prior double valve surgery (mitral repair and aortic replacement), surgical left atrial appendage exclusion, sick sinus syndrome status post permanent pacemaker implant, paroxysmal atrial fibrillation, and severe bioprosthetic aortic stenosis status post recent valve-in-valve TAVR (Evolut FX+ 26-mm in Trifecta 25 mm) 3 weeks prior presented with decompensated heart failure (NYHA functional class III). Physical examination revealed jugular vein distention, grade III/VI holodiastolic murmur, diminished lung sounds, and bilateral pitting edema. Transesophageal echocardiography revealed new findings of a deep-seated TAVR valve, severe circumferential paravalvular aortic regurgitation with multiple jets (Doppler velocity index 0.52, peak gradient 24 mm Hg, peak velocity 2.4 m/s, P1/2T 358 cm/s^2^), and moderate-to-severe mitral valve regurgitation secondary to anterior mitral valve leaflet impingement from the new TAVR valve (Figures 1A to 1E, Video 1). Given these findings, careful shared decision making with the patient and Heart Team ensued. We discussed the possibility of redo surgery, but given his prior open heart surgery, age, and comorbidities, this was deemed a prohibitive risk. Repeat TAVR was discussed; however, there was concern regarding coronary sequestration, future coronary access, and high valve-in-valve-in-valve gradients without addressing the impingement on the mitral valve. An alternative option of valve repositioning followed by reimplantation of another TAVR prosthesis was discussed, and after much evaluation, a shared decision was made with the patient to proceed with this methodology.

**Figure 2:**
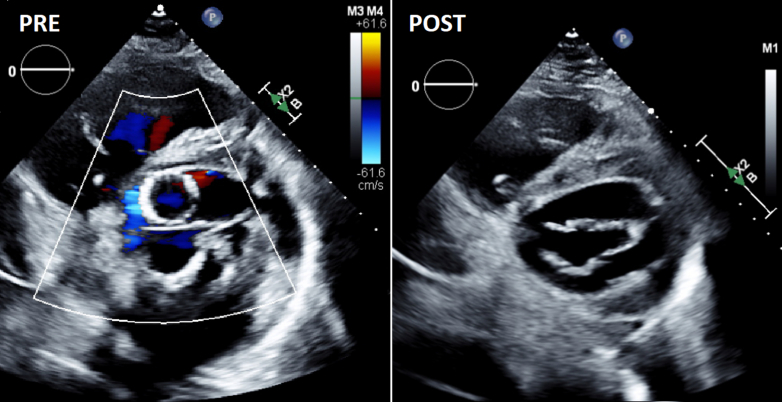
Pre and Post Repositioning Parasternal short-axis views on transthoracic echocardiography revealing the deep transcatheter aortic valve replacement valve with paravalvular leak (Pre) followed by repeat echocardiography demonstrating resolution (Post).

The patient remained hemodynamically stable throughout the duration of the procedure with minimal intermittent aliquots of low-dose norepinephrine to keep mean arterial pressure >65 mm Hg. Post-deployment transthoracic echocardiogram demonstrated successful repositioning of the previously implanted valve (Figure 2) with a well-expanded new TAVR prosthesis with no significant paravalvular leak (mean gradient 17 mm Hg) and only mild mitral regurgitation (Video 2). The patient's hemodynamics stabilized, and he was extubated later that evening. His symptoms fully resolved within hours. He is now 3 months post index procedure and continues to do well with no cardiopulmonary limitations to date.

## Procedural Steps

**Figure 3:**
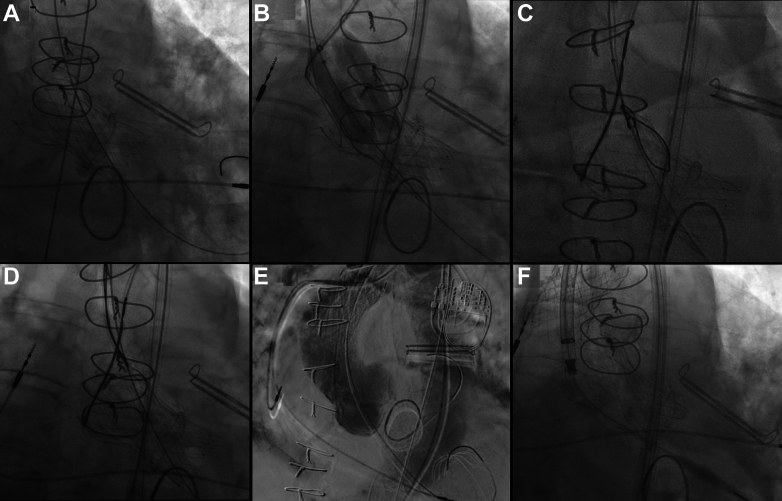
Fluoroscopic Images of Procedural Steps (A) Advancement of the Lunderquist wire. (B) Advancement of the True Flow Balloon. (C) Snaring of the tabs. (D) Gentle back-traction on the snares attached to the valve frame in crisscross fashion. (E) Aortogram confirming successful repositioning of the original transcatheter aortic valve replacement valve. (F) Successful reimplantation of a new 26-mm Evolut FX+ valve.

**Figure 4:**
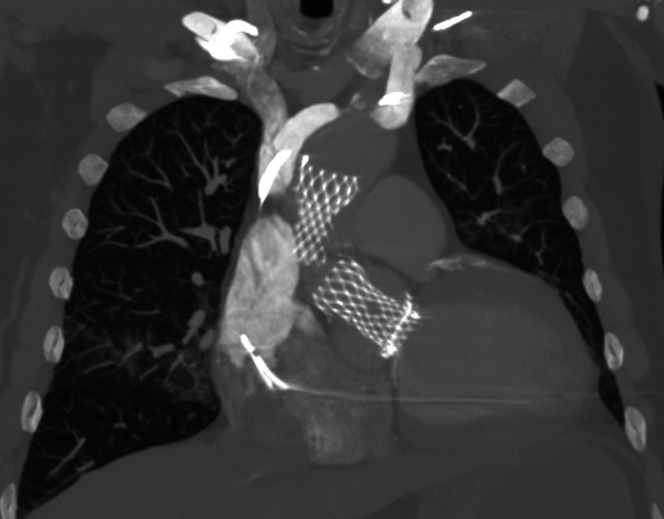
Computed Tomography of Chest Coronal view of the new 26-mm Evolut FX+ transcatheter aortic valve replacement valve implanted status post snare-mediated repositioning of the original valve in ascending aorta distal to the brachiocephalic artery.

Bilateral femoral and right radial arterial accesses were obtained via ultrasound-guided Seldinger technique. Preclosed 12-F and 16-F sheaths were inserted into the left and right femoral arteries, respectively, and a 6-F × 55 cm sheath was inserted radially. We first advanced a Lunderquist Extra-Stiff Double Curve Wire Guide (Cook Medical) across the existing TAVR valve into the left ventricle via the right femoral artery (Figure 3A). Over the Lunderquist, we then advanced a 26 mm × 4.5 cm True Flow balloon (Bard) (Figure 3B). Via the left femoral artery, a 7-F × 45 cm sheath was inserted through which we passed a 6-F multipurpose catheter and a 0.035-inch Glidewire Advantage. After repeated attempts, we were able to wire through the inflated True Flow balloon and pass the multipurpose catheter through it, then deflate the balloon. We repeated this process using a Judkins Right 4 (JR4) catheter from the radial artery with a Glidewire. Once both catheters were through the True Flow balloon, we exchanged them for Two 30-mm Amplatz Goose Neck snares (Medtronic). We then snared the C-tab on the noncoronary cusp side from the left groin and the left coronary cusp side of the TAVR valve from the right arm, securing both in crisscross fashion (Figure 3C). Gradually, we began to pull on the snares simultaneously while inflating the True Flow balloon to maintain blood pressure and avoid aortic trauma (Figure 3D). As we pulled, the valve started to collapse inwardly, and the pressure resulted in the valve dislodging onto the balloon. With the balloon inflated, we were able direct the valve and release it at the desired location in the ascending aorta just distal to the innominate artery. An aortogram confirmed no injury (Figure 3E). We released the left femoral snare. We continued to cinch the C-tab via the right radial artery to hold the valve in place. Over the in-place Lunderquist from the right femoral artery, a new 26 mm Evolut FX+ valve was then deployed in sheathless fashion via standard methodology (Figures 3F and 4). Standard closure techniques were used for all access sites. See Video 3 for compiled cineangiograms, and Supplemental Table 1 for equipment list.

## Potential Pitfalls

### Persistent suboptimal valve positioning

This case highlights the challenges and complexities that can arise with valve-in-valve TAVR procedures. The successful management of this case underscores the importance of meticulous planning, advanced interventional techniques, and shared decision making via a multidisciplinary team approach. Owing to our patient's age, prior surgeries, and comorbidities, we ultimately elected to proceed with percutaneous TAVR valve salvage via intentional valve repositioning followed by reimplantation of another TAVR prosthesis. Although we initially intended to try to reposition the valve within the Trifecta prosthesis, we knew there was a possibility that the valve would need to be released in the ascending aorta if it could not be properly repositioned. This would ensure preservation of future coronary access. It is important to note that repeat TAVR alone would not suffice. Re-do TAVR would result in continued anterior mitral valve impingement with severe mitral regurgitation and may cause acute coronary artery sequestration/occlusion that may have been catastrophic.

### Undesirable device embolization

Valve migration and embolization is generally considered catastrophic and can require urgent surgical intervention, as described by Cribier et al^2^ However, all valve embolizations reported in the TranscatheteR heArt Valve EmboLization (TRAVEL) registry have been, to our knowledge, unintentional and uncontrolled. Despite this, only 19% of these cases required conversion to open heart surgery as their bail-out strategy.^1^ This case demonstrates that intentionally repositioning the TAVR valve in the ascending aorta may be safe, provided that no major arterial branches are obstructed. Few techniques to correct malpositioned TAVR valves have previously been described in the literature, but these are limited to brief reports,^3^, ^4^, ^5^ none of which involved the newer generation Evolut FX+ valve. Ponangi et al^3^ reported a snare technique used to reposition a self-expanding Portico (Abbott) TAVR valve. Saltiel et al^4^ recently demonstrated the use of 2.0-mm rat tooth grasping forceps to rotate an embolized Edwards SAPIEN (Edwards Lifesciences LLC) valve that had descended as far down as the celiac artery. None of these previously reported cases used the True Flow balloon, the first and only flow-through valvuloplasty catheter in the United States and Europe that allows for predilatation of a stenotic aortic valve without clinically significant movement of the device while maintaining adequate blood pressure in the presence or absence of ventricular pacing. The balloon uses a unique 8-chamber inflation design to allow continuous cardiac blood flow through the patent central lumen. The preemptive wiring through the central orifice of a True Flow balloon provided a novel safety mechanism for repositioning the deeply implanted valve. Having a wire across the prosthesis in the left ventricle ensures that the TAVR will not change orientation once detached and allows for a facile repeat TAVR.

### Risk for aortic trauma and conversion to open heart surgery

**Figure 5:**
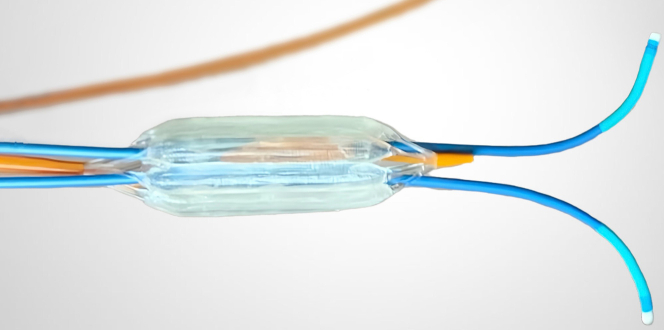
Benchtop Demonstration of Equipment Configuration for Safe Transcatheter Aortic Valve Replacement Valve Repositioning

We recommend inflating the balloon across the deeply implanted TAVR prosthesis to produce a single controlled open orifice, then using directional guides to pass a Glidewire through the balloon. Once the wire is in the left ventricle, it can be exchanged for the snare catheter and a 30-mm Goose Neck snare. This step is repeated twice. A benchtop demonstration of the equipment configuration is displayed in Figure 5.

Once the snares are passed through the balloon, the balloon should be deflated and pulled back to the arch, followed by the snaring of the contralateral C-tabs. This crisscross vector will allow the valve to fold inward to avoid aortic trauma. Once in position to pull on the snares, the distal end of the True Flow balloon should be inflated just above the TAVR valve. With gentle traction, the C-tabs are then pulled off the aortic wall, and with continued traction, the TAVR valve is eventually dislodged. Significant force may be needed depending on how long the TAVR valve has been implanted owing to endothelialization, and a surgical team should be available on standby. As long as the True Flow balloon is inflated, with snares through the central orifice, the repositioned valve will not move past the distal end of the balloon and will be brought toward the balloon rather than inadvertently into the aortic wall.

### Snare and prosthesis entanglement

Thereafter, only one of the snares should be released while the other holds the valve in place. We recommend releasing the snare on the inner curvature of the aorta to avoid any entanglement with the new TAVR prosthesis.

## Conclusions

The key factors for successful strategic repositioning are to ensure a controlled setting, to maintain guidewire access through the valve to prevent it from rotating on itself, and to use a double-snare crisscross technique to improve stability by securing both the inner and the outer valve components. Additionally, the use of a True Flow balloon, which has central patency when inflated, can enhance control over valve maneuverability and repositioning while providing a backstop when pulling on the snares, mitigating the risk of aortic trauma and unintentional pullback across the aortic arch. Nevertheless, these techniques are associated with high risk, including potential complications such as aortic trauma and stroke. These cases open the doors of discussion as to how the repositioning of a TAVR valve or even its embolization can be rendered relatively harmless and, in our case, even strategic. Large-scale studies and further follow-up are necessary to better assess the long-term performance of the extraction and reimplantation strategy described herein.

### Data Availability Statement

The data involved in this case study are available from the authors on request.

**Equipment List:** 

Equipment	No. Used
CATHETER ANGIO 5FR L100CM GRY S STL NYL JR4 3 SEG BRAID L	1
CATHETER BALLOON DIL VALVULOPLASTY 16 FRX110 CM 26 MMX35 CM	2
CATHETER COR DIAG PIGTAILS PIG 145 CRV 5FR 110CM 6 SIDE H	1
CATHETER GUID 6FR DIA0.071IN SHFT NYL STD L JR 4 CRV ENH	1
CATHETER GUID 6FR L100CM DIA0.071IN NYL SHFT AL1.0 W/O SIDE	1
CATHETER GUID 6FR L100CM ID0.071IN COR MP 1 NYL L LUMN MID	1
DEVICE CLOSURE PERCLOSE PROSTYLE	3
DEVICE CLSR 8FR 0.038IN VASC V TWST INTEGR PLATFRM	2
DRAPE SURG NEO W43.5XL60IN ABSRB REINF W18XL20IN FEN	3
GUIDEWIRE VASC L150CM DIA0.035IN FLX TIP L7CM PTFE STR FIX	1
GUIDEWIRE VASC L150CM DIA0.035IN NIT HYDRPHLC TAPR STD ANG	1
GUIDEWIRE VASC L190CM MICROGLIDE COAT STR RADPQ SHP ATRAUM	1
GUIDEWIRE VASC L260CM DIA0.035IN COIL L15CM FLX TIP L4CM	1
GUIDEWIRE VASC L260CM DIA0.035IN L7CM DIA3MM J TIP PTFE S	1
GUIDEWIRE VASC L260CM DIA0.035IN TAPR L11CM FLPY TIP L4CM	1
GUIDEWIRE VASC L260CM DIA0.035IN TIP L5CM PERIPH NIT	1
GUIDEWIRE VASC L300CM MICROGLIDE COAT STR RADPQ SHP ATRAUM	1
INTRODUCER BLLN 12FR L30CM DIA4MM 0.038IN CLS NONTAPERED	1
INTRODUCER CATH 6FR L45CM GWIRE 0.038IN W/ SM CK FLO VLV	1
INTRODUCER SHTH STIFF 4 FRX9 CM 7 CM SET NIT MICRO-STICK	1
KIT ANGIO W/ AT P65 PREM HND CTRL FOR CNTRST DEL ANGIOTOUCH	1
KIT MONITR SING MEDEX LOGICAL 60IN	1
KIT SNR L120CM LOOP DIA30MM CATH 6FR 90DEG G PLT TUNGSTEN	3
PACK SURG CUST CARDIAC CATH LAB PACK SURG CUST LF LIMA	2
SHEATH GUID 6FR L55CM DIA2.2MM GWIRE 0.038IN RAABE RADPQ	1
SHEATH GUID 6FR L90CM ID2.2MM 0.038IN TO INTRODUCE BLLN CLS	1
SHEATH INTRO 10FR L10CM DIL L2.5CM PERIPH W/ MINI S STL SPR	2
SHEATH INTRO 16FR L33CM OD6.1MM ID5.3MM HYDRPHLC KINK	1
SHEATH INTRO 6FR L10CM MINI GWIRE L45CM 0.035IN COR KINK	1
SHEATH INTRO 6FR L10CM NDL 21GA L38MM DIL 0.021IN NIT FLPY	1
SHEATH INTRO 7FR L45CM NYL PTFE S STL HYDROPHILLIC STR CRSS	1
SYSTEM DELIVERY 23-29 MM EVOLUT FX	1
SYSTEM LOADING 23-29 MM EVOLUT FX	1

## Funding Support and Author Disclosures

The authors have reported that they have no relationships relevant to the contents of this paper to disclose.

## Appendix

Video 1 Deep-Seated TAVR Valve With Severe Paravalvular Leak Transesophageal echocardiography revealing new findings of a deep-seated transcatheter aortic valve replacement valve, severe circumferential paravalvular aortic regurgitation with multiple jets, and moderate-to-severe mitral valve regurgitation secondary to anterior mitral valve leaflet impingement.

Video 2 Post-Redeployment Transthoracic Echocardiography Successful repositioning of the previously implanted valve with a well-expanded new transcatheter aortic valve replacement prosthesis with no significant paravalvular leak and only mild mitral regurgitation

Video 3 Compiled Cineangiograms

Supplemental Table 1 Equipment List
