# Supplementary material for: Salvaging Percutaneously a Suboptimal TAVR Deployment with Repositioning In the Thoracic Aorta: A Novel Snare-Mediated Approach to Prosthesis Malposition
Source: JACC Case Rep. 2025 Jul 18;30(24):104553. doi: 10.1016/j.jaccas.2025.104553 (PMC12371381; doi:10.1016/j.jaccas.2025.104553)
Supplement: Supplemental Table 1 — Equipment List [file mmc4.docx]

**Supplemental Table 1: Equipment List**

| Equipment | No. Used |
| --- | --- |
| CATHETER ANGIO 5FR L100CM GRY S STL NYL JR4 3 SEG BRAID L | 1 |
| CATHETER BALLOON DIL VALVULOPLASTY 16 FRX110 CM 26 MMX35 CM | 2 |
| CATHETER COR DIAG PIGTAILS PIG 145 CRV 5FR 110CM 6 SIDE H | 1 |
| CATHETER GUID 6FR DIA0.071IN SHFT NYL STD L JR 4 CRV ENH | 1 |
| CATHETER GUID 6FR L100CM DIA0.071IN NYL SHFT AL1.0 W/O SIDE | 1 |
| CATHETER GUID 6FR L100CM ID0.071IN COR MP 1 NYL L LUMN MID | 1 |
| DEVICE CLOSURE PERCLOSE PROSTYLE | 3 |
| DEVICE CLSR 8FR 0.038IN VASC V TWST INTEGR PLATFRM | 2 |
| DRAPE SURG NEO W43.5XL60IN ABSRB REINF W18XL20IN FEN | 3 |
| GUIDEWIRE VASC L150CM DIA0.035IN FLX TIP L7CM PTFE STR FIX | 1 |
| GUIDEWIRE VASC L150CM DIA0.035IN NIT HYDRPHLC TAPR STD ANG | 1 |
| GUIDEWIRE VASC L190CM MICROGLIDE COAT STR RADPQ SHP ATRAUM | 1 |
| GUIDEWIRE VASC L260CM DIA0.035IN COIL L15CM FLX TIP L4CM | 1 |
| GUIDEWIRE VASC L260CM DIA0.035IN L7CM DIA3MM J TIP PTFE S | 1 |
| GUIDEWIRE VASC L260CM DIA0.035IN TAPR L11CM FLPY TIP L4CM | 1 |
| GUIDEWIRE VASC L260CM DIA0.035IN TIP L5CM PERIPH NIT | 1 |
| GUIDEWIRE VASC L300CM MICROGLIDE COAT STR RADPQ SHP ATRAUM | 1 |
| INTRODUCER BLLN 12FR L30CM DIA4MM 0.038IN CLS NONTAPERED | 1 |
| INTRODUCER CATH 6FR L45CM GWIRE 0.038IN W/ SM CK FLO VLV | 1 |
| INTRODUCER SHTH STIFF 4 FRX9 CM 7 CM SET NIT MICRO-STICK | 1 |
| KIT ANGIO W/ AT P65 PREM HND CTRL FOR CNTRST DEL ANGIOTOUCH | 1 |
| KIT MONITR SING MEDEX LOGICAL 60IN | 1 |
| KIT SNR L120CM LOOP DIA30MM CATH 6FR 90DEG G PLT TUNGSTEN | 3 |
| PACK SURG CUST CARDIAC CATH LAB PACK SURG CUST LF LIMA | 2 |
| SHEATH GUID 6FR L55CM DIA2.2MM GWIRE 0.038IN RAABE RADPQ | 1 |
| SHEATH GUID 6FR L90CM ID2.2MM 0.038IN TO INTRODUCE BLLN CLS | 1 |
| SHEATH INTRO 10FR L10CM DIL L2.5CM PERIPH W/ MINI S STL SPR | 2 |
| SHEATH INTRO 16FR L33CM OD6.1MM ID5.3MM HYDRPHLC KINK | 1 |
| SHEATH INTRO 6FR L10CM MINI GWIRE L45CM 0.035IN COR KINK | 1 |
| SHEATH INTRO 6FR L10CM NDL 21GA L38MM DIL 0.021IN NIT FLPY | 1 |
| SHEATH INTRO 7FR L45CM NYL PTFE S STL HYDROPHILLIC STR CRSS | 1 |
| SYSTEM DELIVERY 23-29 MM EVOLUT FX | 1 |
| SYSTEM LOADING 23-29 MM EVOLUT FX | 1 |
